# Part IV. Miscellanies

**Published:** 1833-07-01

**Authors:** 


### Miscellanies.

### I. PERIODICAL PRESS.

The desire of R. B. S. to give our readers "an accurate review, or short description of the Medical Periodical Press, as it at present exists, or has existed, since the year 1800,"—is a task which could not be reasonably or fairly undertaken by us. We do not see the lives of men published till after their death—and therefore we could not possibly offer any critical notices of contemporary periodicals. Our correspondent complains that, in remote parts of the country, medical readers are often puzzled by references to periodical journals, of which they are ignorant, and wishes to know what are the actual existing medical journals of the present day. This list we can have no objection to furnish, according to seniority.

1. The LONDON MEDICAL and PHYSICAL JOURNAL, which commenced in March, 1799, and still continues in its monthly form. This is, of course, the father of existing periodical journals of medicine in this country. It has attained its 68th volume.

2. The EDINBURGH MEDICAL and SURGICAL JOURNAL, *quarterly*, commenced in January, 1805, and still continues, having attained its 28th year.

3. The "NEW MEDICAL and PHYSICAL JOURNAL," which commenced in a *monthly* form, in 1811, and continued till January, 1816, when it changed its title to "The MEDICO-CHIRURGICAL JOURNAL and REVIEW," conducted by Drs. Shearman, Johnson, and Palmer. The work continued in its *monthly* form till July, 1818, when it became the property of Dr. Johnson, who changed it into a *quarterly* periodical, under the title of the "MEDICO-CHIRURGICAL REVIEW, &c." which it still retains.

4. The LONDON MEDICAL REPOSITORY, which commenced in 1814, as a monthly journal, and still continues, but now in a *weekly* form, under the title of the "LONDON MEDICAL and SURGICAL JOURNAL."

5. The LANCET, a weekly Journal, which commenced in the Autumn of 1823, and still continues.

6. The LONDON MEDICAL GAZETTE, which commenced in December, 1827, as a weekly publication, still continues.

7. The GLASGOW JOURNAL of MEDICINE and SURGERY, is the oldest of the English provincial periodicals, and, indeed, we may say, the only one now in existence.

8. The DUBLIN JOURNAL of MEDICAL and CHEMICAL SCIENCE, has lately started, and is well known to our readers, by the extracts which we give from this our respected contemporary.

Thus then, we see that, at the beginning of the present century there was but *one* periodical journal in our profession; and now there are EIGHT! If this be not a proof of the "*march of intellect*," we know not what is! We have not touched on the many journals which have started during the last 33 years, and which are now no more. "De mortuis nil." As to our living contemporaries, there is not one which we wish to see extinct—not one against which we harbour the slightest feeling of envy, hatred, or malice. Content with our own success, we wish a greater degree of it to every one of them. We hope yet to see the day, when that courtesy and decorum which ought to exist among members of a liberal and enlightened profession, will also exist among periodical journalists. We do not attempt to absolve ourselves from the sin and shame of having entered into contentions and collisions; but we trust that, in accordance with the spirit of the times, we have REFORMED. Sincerely do we hope that our contemporaries will pursue the same course.

---

### II. Remarkable Cessation of the Pulse in both Arms. By Dr. Fergusson, Assist.-Surgeon 5th Regt.

Private John Mathews, 6th Regt. of Foot, aged 24, a delicate young man of leuco-phlegmatic temperament, by trade a tailor, has been several times in hospital, with inflammation of both testicles and tonsils, brought on simultaneously by exposure to wet or cold.

Was sent on escort duty from Abington to Trinicrig, on the 3d of August, 1832, got thoroughly drenched with rain, and on his return was seized with violent pneumonia, which was characterized by distressing palpitation of the heart and a *total absence of pulse in either of his arms or wrists,* though the pulsation of the carotids appeared stronger than usual. The inflammatory symptoms were removed by the usual means, but *this anomalous symptom still continues.*

The chest sounds well on percussion and, on applying the stethoscope, the respiratory murmur is distinctly audible in all parts, but is accompanied by a subcrepitating râle in the left side of chest; the heart's pulsation is quick and regular, but weak, and can be felt under the margin of the 7th rib. Some months since there was a pulsation under the upper third of the sternum, which is not now audible. The palpitation is always worse at night, and is accompanied by a peculiar *noise,* which deprives him of all rest; he is also frequently seized with a shooting pain under the sternum and in the region of the heart, which is quite intolerable: this always attacks him at night, and is attended with a feeling of suffocation and obstructed circulation to the head; the face becomes rather livid, and the eyes tinged; he can only partake of spoon or milk diet, but he does not reduce in flesh, yet he is incapable of the slightest exertion.

☞ Dr. F. requests our opinion on the above case; but we prefer waiting till next quarter, when the issue will probably be known.

---

### III. Cholera.

We are induced once more to suspend the publication of our Excerpta Choleralogica, since the subject seems to have dropped entirely from men's minds for some months past. If it be the specific contagious disease which some of our contemporaries have designated it, we will surely have the disease again in the course of the Summer or Autumn, and then we shall resume our Excerpta. By the way, we have lately seen three or four cases of cholera precisely similar to even the bad cases of last year. None of them, however, proved fatal.

---

We have received the letter of an "Apothecary," reflecting on a gentleman who has lately written on medical politics in the Sister Isle. We can have nothing to do with men's private affairs, but only with the books which they may usher before the public.

We have been favoured with a view of a Lithographic Plate of Inguinal Hernia, about to be published, with one of Femoral Hernia, by Mr. Bloxam, of Hanover-Street. It is on the plan of Layers, and depicts the Fasciæ, apertures, &c. with equal accuracy and clearness. It would be very useful to the student.

### IV. Medical Politics.

#### Apothecaries.

A considerable excitement has been raised in more than one branch of the profession by the recent proceedings in Parliament. Before this Journal sees the light, the question will probably be decided, not quite to the satisfaction of any party. It is almost needless for us to make any remarks under such circumstances, and therefore we shall be very brief. And first, in respect to the graduates of Edinburgh. If such graduates choose to practise physic in England, and dispense their own medicines, it is monstrous that they should be obliged to undergo an examination in physic before a tribunal of London Apothecaries! The production of their diploma before the Court of Examiners ought to be a sufficient guarantee of their competency to practise physic in any way they please, and the licence should be immediately granted them, on paying a small sum of money, and undergoing an examination in pharmacy. We think it quite necessary that they should present themselves, with their diplomas, before the Company, in order that they may be enrolled as regular and certified practitioners, otherwise the Company will find it difficult to prosecute unqualified practitioners, not knowing whether or not they may hold a diploma. Nearly the same line of argument ought, we think, to hold good respecting members of the Dublin and Edinburgh Colleges of Surgery. The production of their diplomas ought to entitle them to a recognition, or, at all events, to an examination, not only before the Court of Examiners of the Apothecaries' Company, but of the Royal College of Surgeons. It is nonsense to say that England will be swamped with Scotch practitioners, if such facilities be allowed. Neither will these facilities induce English youths to go to Scotland to acquire their medical education, unless an obstinate adherence to the APPRENTICESHIP-SYSTEM drives a certain number beyond the Tweed, to escape the drudgery and degradation of five years behind the counter, like a cheese-monger's fag! We have reason to believe that some such regulations as the above will be agreed upon by the framers of the Bill and the opposers of it. But we earnestly exhort the medical profession throughout the whole kingdom to petition the legislature, without delay, for a Parliamentary inquiry into the general state of the medical profession in these Isles. Nothing short of such an inquiry will be productive of any lasting benefit, or secure any liberal measure.

---

